# Protein Contents Determine the Thermal Stability and Gel Consistency of High-Amylose Milled Rice

**DOI:** 10.3390/foods14244353

**Published:** 2025-12-18

**Authors:** Yizhang Feng, Yandong Huang, Zhongquan Cai, Shuolei Liao, Shahzad Ahmad, Xiaokun Huang, Jiangchuan Li, Xiaochen Qi, Yuning Wu, Zhenzhou Wu, Piqing Liu, Yongfu Qiu

**Affiliations:** 1 State Key Laboratory of Conservation and Utilization of Subtropical Agro-Bioresources, College of Agriculture, Guangxi University, Nanning 530005, China; 2317301010@st.gxu.edu.cn (Y.F.); 2317301018@st.gxu.edu.cn (Y.H.); 2117301026@st.gxu.edu.cn (S.L.); shahzadahmadpbg@gmail.com (S.A.); 2231200204@st.gxu.edu.cn (X.H.); 2317301022@st.gxu.edu.cn (J.L.); 2317393033@st.gxu.edu.cn (X.Q.); 2417301053@st.gxu.edu.cn (Y.W.); 2417391076@st.gxu.edu.cn (Z.W.); 2 Guangxi Key Laboratory of Agro–Environment and Agro–Products Safety, Key Laboratory of Crop Cultivation and Physiology, Education Department of Guangxi Zhuang Autonomous Region, College of Agriculture, Guangxi University, Nanning 530004, China; 3 Institute for New Rural Development, National Demonstration Center for Experimental Plant Science Education, Guangxi University, Nanning 530004, China; zq_cai@163.com

**Keywords:** high-amylose rice, protein content, pasting properties, gel consistency, thermal stability, starch-protein interaction

## Abstract

Protein and starch are the two primary components of rice flour, significantly influencing their gelatinization and gel consistency. However, the role of protein in the gelatinization properties and gel consistency of high-starch starch remains unclear. Our study investigated the influence of protein on the gelatinization and gel consistency of high-amylose rice flour by analyzing six high-amylose rice varieties with differing protein levels. The results demonstrated that elevated protein content was associated with reduced breakdown (BD) and gel consistency. Additionally, a recombinant rice flour (RRF) system was developed by reintroducing extracted proteins into high-amylose rice flour in various ratios. The findings indicated that increasing protein proportions in the RRF system led to a marked decrease in gel consistency, accompanied by reductions in peak viscosity (PV), BD, final viscosity (FV), and setback (SB), while peak time (PeT) and pasting temperature (PaT) exhibited significant increases. Correlation analysis and microstructure observations support the hypothesis that proteins may enhance the stability of the paste by restricting the expansion of starch granules during gelatinization, which is related to the reduction in gel consistency. This study confirmed that protein content plays a significant role in determining the gel consistency of high-amylose rice, guiding the improvement of the quality of use or cultivating high-amylose rice suitable for processing.

## 1. Introduction

Rice (*Oryza sativa* L.) is the staple food for more than half of the world’s population and plays a crucial role in global food security [1]. With the improvement of living standards, consumers’ demands have shifted towards consuming rice of a better quality and that is beneficial to health [2]. The quality of rice is mainly defined by the interaction between protein and starch during the heating process, so these components are crucial for understanding its cooking and eating characteristics [3,4]. High-amylose rice has attracted extensive attention due to its firm texture, slow digestibility and stable gelatinization characteristics, making it valuable for both consumer and functional food applications [5,6]. Conventionally, studies on rice have focused on the role of starch composition, particularly amylose content, in shaping gelatinization behavior and gel consistency [7]. This starch-centric perspective has guided most quality assessment systems and breeding decisions [8,9,10,11].

However, rice flour is a complex system in which protein plays a crucial but often underestimated role. During the process of heating and gelatinization, proteins form a physical barrier around the starch granules, restricting their water absorption, expansion, and rupture [12,13,14]. The interaction between these proteins and starch can significantly alter the gelatinization characteristics of starch and the final gel texture [15,16,17]. Although previous studies have emphasized the role of protein content in waxy or medium to low amylose content rice [18,19], its precise contribution to high-amylose systems remains unclear. Research on the influence of protein on the gelatinization characteristics and gel consistency of rice flour with high amylose content is lacking, necessitating further investigation.

Therefore, in order to clarify how protein content affects the gelatinization characteristics and gel consistency of high-amylose rice, we analyzed six varieties with similar amylose content but different protein levels to clarify the relationship between protein content and the gelatinization characteristics and gel consistency of high-amylose rice. The role of proteins was isolated and verified using the recombinant rice flour (RRF) model. Our experimental results show that the increase in protein content is closely related to the decrease in gel consistency and the change in gelatinization characteristics of high-amylose rice. Through correlation analysis and microscopic observation, we speculate that the protein content affects the gel consistency by altering the thermal stability of the rice flour. Our research results provide important guidance for the precise breeding of high-amylose rice and the production of special rice for processing.

## 2. Materials and Methods

### 2.1. Materials

Six high-amylose indica rice varieties (Zhengui’ai, Guichao 2, N5088, V5213, V4233, and Yufengzhan) were cultivated during the late growing season of 2024 in the experimental fields of Guangxi University. After harvest, the grains were dried, milled, and polished to obtain milled rice for further analysis.

### 2.2. Major Equipment and Reagents

Main reagents: Sodium hydroxide pellets (≥99%), potassium hydroxide pellets (≥85%), hydrochloric acid (37%, analytical grade), anhydrous ethanol (≥99.5%, analytical grade), and bromothymol blue were purchased from Tianjin Damao Chemical Reagent Factory (Tianjin, China).

Instruments Used: High-Speed Grinder (800Y, Yongkang Botou Hardware Products Co., Ltd., Yongkang, China), UV Spectrophotometer (Cary 60, Agilent Technologies Trading Co., Ltd., Santa Clara, CA, USA), Rapid Visco Analyzer (RVA 4500, Perten, Stockholm, Sweden), Rice Taste Quality Analyzer (JSWL, Beijing Dongfu Jiuheng Instrument Technology Co., Ltd., Beijing, China), High-speed refrigerated centrifuge (5810R, Eppendorf AG, Enfield, CT, USA), and Scanning Electron Microscope (FEI Quattro S, Thermo Electron Corporation, Waltham, MA, USA).

### 2.3. Determination of Proximate Composition and Gel Consistency of Milled Rice

Samples from the said varieties were ground into powder using a High-Speed Grinder and passed through a 100-mesh sieve for subsequent analysis. Total starch content was determined using a starch assay kit (Solarbio, Beijing, China, Cat. No. BC0700) according to the manufacturer’s instructions via UV spectrophotometry. Amylose content was measured by iodine colorimetry [20]. Amylopectin content was calculated by subtracting the amylose content from the total starch content. Crude fat content was determined by Soxhlet extraction using petroleum ether [21]. Gel consistency was measured according to GB/T 17891-2017 “High-Quality Rice Grains” [22].

Pasting properties were analyzed using a Rapid Visco Analyzer. Briefly, 3 g of rice flour was mixed with 25.0 mL of deionized water and tested according to GB/T 24852-2010 [23]. Data were processed using TWC software(TWC 4.0).

Protein content was determined with a Rice Taste Quality Analyzer based on near-infrared spectroscopy. For each variety, 150 g of milled rice was analyzed in triplicate using the indica rice mode.

### 2.4. Preparation of Rice Protein and Starch

Rice protein and starch were prepared according to the method described by Zhao et al. (2022) with minor modifications [19]. The rice powder was defatted by mixing with anhydrous ethanol at a 1:4 (*w*/*v*) ratio. The defatted rice powder was air-dried at room temperature for 24 h, then mixed with 0.05 M NaOH solution at a 1:4 (*w*/*v*) ratio and stirred at 250 rpm for 3 h at room temperature. The mixture was centrifuged at 7500 rpm/min for 15 min, and the supernatant was collected. This extraction process was repeated three times. After collecting all supernatants, the pH was adjusted to 4.8 using 0.1 M HCl, and the solution was allowed to stand at room temperature for 1 h to precipitate the proteins. The precipitated proteins were collected by centrifugation at 7500 rpm/min for 20 min. The resulting pellet was freeze-dried. The rice starch obtained after alkaline extraction and centrifugation was washed with deionized water until a neutral pH was achieved and then freeze-dried at −40 °C. The dried protein and starch samples were stored at −20 °C for subsequent use.

### 2.5. Preparation of Reconstituted Rice Flour from Different Varieties

Reconstituted rice flour (RRF) was prepared by mixing extracted starch from each variety with its corresponding protein at 0%, 5%, and 10% (*w*/*w*) levels. All starches and proteins used for the preparation of RRF were separated and extracted in accordance with the method in Section 2.4.

### 2.6. Characterization of Gel Consistency and Pasting Properties of RRF

The gel consistency of the RRF was determined according to GB/T 17891-2017 “High-Quality Rice Grains” [22]. Pasting properties were analyzed using a Rapid Viscosity Analyzer. Briefly, RRF samples, formulated at protein supplementation levels of 0%, 5%, and 10% (*w*/*w*), were prepared by homogenizing 3.0 g of flour in 25.0 mL of deionized water on a shaker (250 rpm, 1 h, room temperature). The resulting suspension was then subjected to RVA analysis, and the data were processed with the accompanying TWC software.

### 2.7. Microstructural Analysis by Scanning Electron Microscope

The gel microstructure was analyzed by scanning electron microscope (SEM) according to a previously established method [19]. In detail, RRF (2.5 g) was hydrated in 5 g of deionized water and homogenized by shaking at 240 rpm for 1 h at room temperature. The mixture was then gelatinized in a water bath at 95 °C for 15 min. The gels were cooled to room temperature, frozen at −80 °C for 24 h, and subsequently freeze-dried at −40 °C. The freeze-dried samples were sectioned into small pieces using a stainless-steel knife, mounted on sample holders, sputter-coated with gold to ensure conductivity, and observed under SEM at a magnification of 2000×.

### 2.8. Statistical Analysis

All experiments were independently repeated at least 3 times, and the data are presented as averages. The Shapiro–Wilk test was employed to assess the normality of the data distribution. One-way analysis of variance (ANOVA) was utilized to evaluate differences among multiple means, followed by Duncan’s multi-range test for post hoc comparisons (*p* < 0.05). The relationship between components and functional attributes was assessed using Spearman’s rank correlation analysis. The intensity of correlations is categorized as follows: r ≥ 0.7 (strong), 0.5–0.7 (moderate), and <0.5 (weak). The significance levels are indicated as follows: * *p* < 0.05, ** *p* < 0.01, and *** *p* < 0.001, denoting statistically significant results. All statistical analyses were performed using SPSS 26.0 (IBM, Armonk, NY, USA), and graphics were generated with OriginPro 2024 (OriginLab, Northampton, MA, USA).

## 3. Results

### 3.1. Association Between Protein Content and Gel Consistency in High-Amylose Milled Rice

The proximate composition and gel consistency of the six high-amylose rice cultivars are summarized in Table 1. Under the condition of similar amylose content among the tested varieties, protein content emerged as the predominant factor governing gel consistency.

Our test results show that the consistency of the gel continuously decreases as the protein content increases. Among the six varieties, Yufengzhan has the lowest protein content, at 7.0%, but its gel consistency is the longest, at 88 mm. In contrast, Guichao 2 has the highest protein content (9.1%), and its gel consistency is the hardest among the six varieties (27 mm). This indicates that among the six cultivars we tested, proteins play a major regulatory role in adjusting the gel consistency of high-amylose rice. Meanwhile, by analyzing the relationship between the change in crude fat content and gel consistency, we further observed that the crude fat content was negatively correlated with the gel viscosity, and the gel consistency decreased with the increase in crude fat content. The variation trend of gel consistency with protein content is the same as that with crude fat content; this might suggest that there could be a synergistic effect between protein and crude fat. In contrast, total starch, amylose, and amylopectin contents showed limited variation and no clear correlation with gel consistency under the conditions tested.

### 3.2. Protein Content Enhances Pasting Thermal Stability in High-Amylose Rice

The pasting properties of the six high-amylose rice varieties with varying protein contents were analyzed using a Rapid Visco Analyzer (RVA), with key parameters summarized in Table 2.

By comparing the gelatinization characteristic test indicators of the six test varieties, we found that the breakdown (BD) values of the six varieties changed sharply and decreased significantly with increasing protein content. The BD is an indicator characterizing the stability of starch paste under high-temperature shear action. The lower its BD value, the stronger the stability of the starch paste. Among our tested varieties, Guichao 2 (9.1%), which has the highest protein content, had the lowest BD value of 622 cP, while Yufengzhan (7.0%), which has the lowest protein content, had the largest BD value of 1292 cP. This negative correlation between protein content and BD value indicates that protein is an important factor regulating the stability of starch paste during the heating process. The stability induced by this protein was further confirmed by peak time (PeT) and pasting temperature (PaT). High-protein varieties Guichao 2 and N5088 will take a longer time to reach peak viscosity (PV). Their PeT values were 6.64 min and 6.87 min, respectively. N5088 still requires a higher temperature to start pasting, reaching 83.48 °C. In addition, we also observed that there were significant differences in PV, FV and SB among different varieties, and these differences were clearly not related to protein content. This indicates that, apart from protein content, other factors such as the fine structure of starch and the chain length distribution of starch can also affect the gelatinization characteristics of starch.

### 3.3. Protein Content Directly Regulates Gel Consistency in RRF

To clarify the influence of protein on the consistency of the gel consistency. We adopted the RRF method to re-add the separated protein to the starch extracted from each variety, with the re-addition amounts being 0%, 5% and 10% of the total mass, respectively, and measured its gel consistency.

**Figure 1:**
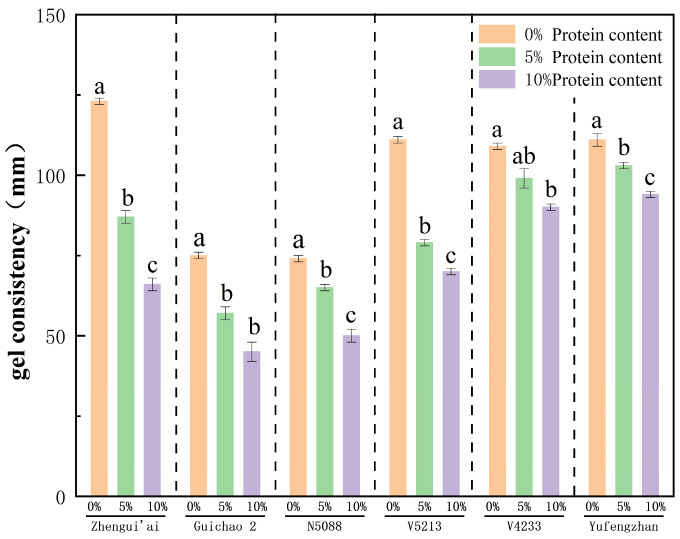
Effect of protein addition level (0%, 5%, 10% *w*/*w*) on the gel consistency of reconstituted rice flour (RRF) from six high-amylose varieties. Within the same group, gel consistency values at different protein levels that differ by lowercase letters are significantly different (*p* < 0.05).

As shown in Figure 1, we observed that the gel consistency of all RRF systems decreased with the increase in protein supplementation. This effect is most obvious among the precious and beloved varieties. When the protein content increased from 0% to 10%, the gel texture dropped from 122 mm to 66 mm. In contrast, the V4233 system has undergone the least change, with a reduction in only 19 mm (from 109 mm to 90 mm). Although there was no statistically significant difference in the gel consistency of the V4233 RRF system when the protein refill was 5% compared with that at the 0% and 10% levels, the overall trend of the gel consistency of all varieties of RRF indicated a decrease with the increase in protein content.

It is worth noting that different varieties of gel consistency have varying sensitivities to protein supplementation. This may indicate that the ability of proteins to reduce viscosity could also be influenced by starch structure or subtle differences in protein architecture.

### 3.4. Thermal Stability Enhancement of RRF by Protein Addition

To understand the influence of protein content on the properties of starch paste, we adopted the RRF method to re-add the separated protein to the starch extracted from each variety, with the re-addition amounts being 0%, 5% and 10% of the total mass, respectively. We systematically analyzed the influence of protein on the gelatinization characteristics of starch. As shown in Table 3, proteins have a significant regulatory effect on the gelatinization characteristics of RRF, among which the change in breakdown (BD) value is particularly prominent.

With increasing protein replenishment from 0% to 10%, the BD values of all recombinant rice flour systems decreased significantly. The BD value of Zhengui’ai, showing greater variation, decreased from 1982 cP to 248 cP, while that of Yufengzhan, exhibiting lesser variation, decreased from 1329 cP to 258 cP. This reduction in BD values may indicate that the protein effectively enhances the structural stability of starch granules under high-temperature shear, thereby improving the thermal stability of the gelatinised system.

The increase in protein content simultaneously leads to rises in peak time (PeT) and pasting temperature (PaT). The PeT and PaT of the 10% protein addition group were significantly higher than those of the low-protein group. Among them, the PeT of 5213 increased from 4.64 min to 6.87 min, and the PaT of 5088 increased from 74.38 °C to 88.33 °C. As the proportion of protein increased, the peak viscosity (PV), final viscosity (FV), and setback (SB) of each system decreased significantly. In addition, the trough viscosity shows a trend of first increasing and then decreasing with the increase in protein content. The differences in gelatinization characteristics among recombinant systems of different varieties with the same protein refill level may stem from the inherent differences in content and structure between amylopectin and amylose.

### 3.5. Scanning Electron Microscopy Reveals Protein-Induced Microstructural Changes in Gels

**Figure 2:**
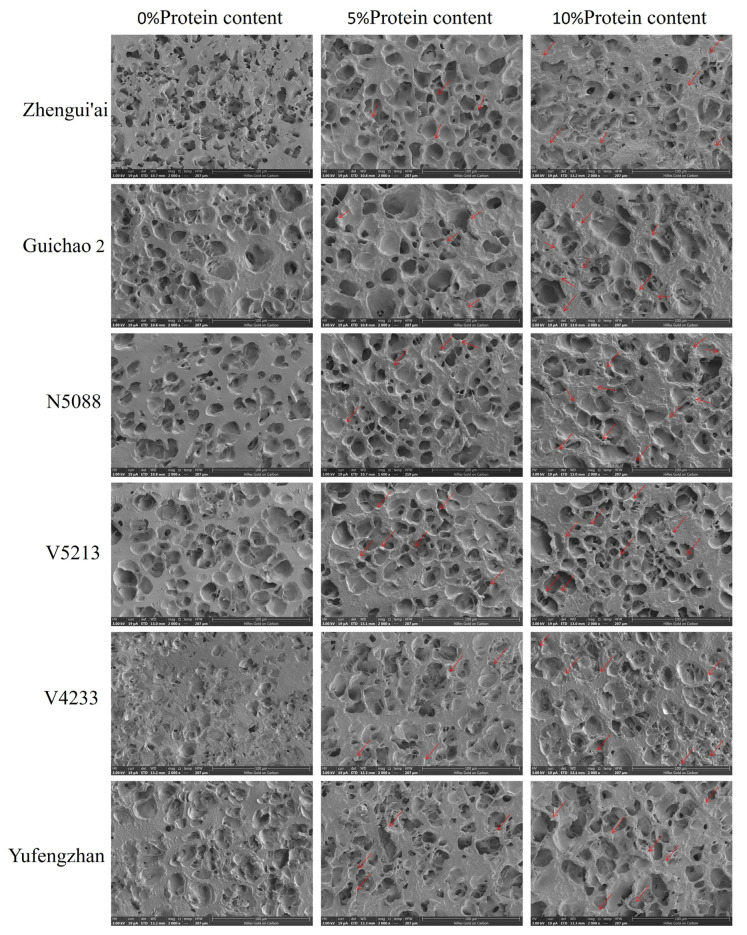
Scanning electron micrographs (2000×) of reconstituted rice flour (RRF) gels with 0%, 5%, and 10% protein addition. Arrows indicate ungelatinized starch granules in the 5% and 10% protein gel.

To observe the effect of proteins on the recombinant rice flour gel network, we used a scanning electron microscope to examine the microstructure of the RRF gel. The results are shown in Figure 2. All the gels formed a honeycomb-like structure. With the increase in the amount of protein refilling, we observed significant changes in the gel structure. When the protein refilling is 0%, the gel shows a continuous and dense structure, and almost no ungelatinized starch particles can be observed. As the protein refilling increases, the gel structure becomes looser and more fragmented, and at the same time, more ungelatinized starch particles can be observed (indicated by the arrow in the figure). These changes might be caused by proteins disrupting the continuity of the starch matrix, resulting in this structural looseness. This directly provides visual evidence and strongly supports our speculation that proteins can restrict the complete expansion and breakage of starch granules during heating. This SEM analysis provided qualitative visual evidence of the microstructural changes in the gel network induced by protein addition.

### 3.6. Correlation Between Major Components of Milled Rice and Tested Parameters

**Figure 3:**
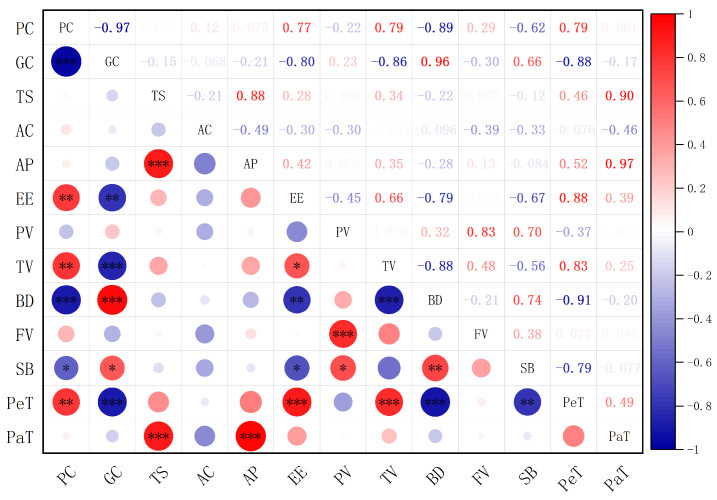
Correlation heatmap between major chemical components and functional properties of high-amylose milled rice. Red and blue represent positive correlation and negative correlation, respectively. The color intensity corresponds to the correlation intensity, and the significance level is indicated by an asterisk (* *p* < 0.05, ** *p* < 0.01, *** *p* < 0.001). PC: Protein content; GC: Gel consistency; TS: Total starch; AC: Amylose content; AP: Amylopectin content; EE: Crude fat content.

In order to investigate the intrinsic relationship between the primary chemical constituents of milled rice and key parameters such as gelatinization characteristics and gel consistency, we systematically analyzed the experimental data using Spearman’s correlation method. The results are presented in Figure 3.

Our relevant new analysis indicates that there is a highly significant negative correlation between protein content and gel consistency (r = −0.97, *p* < 0.001), a highly significant negative correlation with breakdown (BD) (r = −0.89, *p* < 0.001), and a significant negative correlation with setback (SB) (r = −0.62, *p* < 0.05). This indicates that the higher the protein content, the lower the gel consistency, and BD and SB will all decrease. These results indicate that a high protein content significantly reduces the degree of viscosity decomposition at high temperatures. It also reduces the retrograde tendency during the cooling process. This means that the system becomes more stable during the heating process.

Furthermore, protein content was positively correlated with crude fat content (r = 0.77, *p* < 0.01), as well as with trough viscosity (TV) and peak time (PeT) (r = 0.79, *p* < 0.01 for both). This suggests that elevated protein content is not only associated with fat accumulation but also significantly delays the gelatinization process and improves the system’s ability to maintain viscosity during heating. Correlation analysis of crude fat showed a highly consistent trend with protein: crude fat was significantly negatively correlated with both gel consistency and BD (r = −0.80, *p* < 0.01; r = −0.79, *p* < 0.01), significantly negatively correlated with SB (r = −0.67, *p* < 0.05), and positively correlated with PeT and TV (r = 0.88, *p* < 0.01; r = 0.66, *p* < 0.05, respectively). Although the strength of correlation between crude fat and these parameters was slightly lower than that for protein, its regulatory effect on thermal stability during gelatinization appears synergistic with that of protein.

Further analysis of the relationship between gel consistency and other RVA parameters showed that gel consistency was highly significantly negatively correlated with both TV and PeT (r = −0.86, *p* < 0.001; r = −0.88, *p* < 0.001), while it was positively correlated with BD and SB (r = 0.96, *p* < 0.001; r = 0.66, *p* < 0.05, respectively). The strong statistical relationship observed here supports a logical sequence: the higher the protein content, the more restricted the particle expansion (reflected in higher PeT and TV, and lower BD). Conversely, this may affect the quantity and nature of amylose leached during gelatinization, ultimately leading to the formation of a weaker and more fragmented gel network, which is macroscopically manifested as a shorter gel consistency (Figure 1) and is visually supported by SEM observations (Figure 2). It is worth noting that correlation does not imply causation. Therefore, to further confirm the above logic, further experimental verification is needed.

## 4. Discussion

This research systematically elucidates the regulatory role of milled rice protein content in the gelatinization characteristics and gel consistency of high-amylose rice, along with its potential mechanisms, through analyses of six high-amylose rice varieties and starch-protein recombination experiments. Our findings show that protein content is a major factor determining thermal stability, the gelatinization characteristics, and the final gel consistency of high-amylose rice.

### 4.1. Protein Content on Pasting Properties and Thermal Stability

We found that in the natural starch and RRF systems, the breakdown (BD) value continuously decreased with the increase in protein content (Table 2 and Table 3), which was consistent with the results of other starch-protein studies [24]. Meanwhile, in the RRF system, we also observed that as the protein content increased, peak viscosity (PV), final viscosity (FV), and setback (SB) decreased, while peak time (PeT) and pasting temperature (PaT) increased. In addition, perhaps due to the large protein gradient we set, we also found that trough viscosity (TV) first increased and then decreased with the increase in protein content (Table 3). An atypical trend in TV was observed not only in RRF but also in natural rice flour: when the protein content was below 8%, TV increased with rising protein levels; however, once protein content exceeded 8%, TV decreased instead (Table 2). It is worth noting that Guichao 2 has the highest protein content (over 8%). According to our analysis, its TV should be lower than that of Zhengui’ai and N5088. However, in reality, its TV is the highest among the six natural rice flours.

The BD value, a key indicator of starch paste tolerance under high-temperature shear, directly reflects the thermal stability of the system [25,26]. We comprehensively analyze and speculate that the increase in protein content leading to a decrease in BD value might be caused by these two mechanisms: First, competition between protein and starch for available water, limiting granule hydration and expansion [19,27,28,29]. Secondly, the potential formation of a protein network or adsorption onto granule surfaces creates a physical barrier to swelling [30,31]. These two effects work together to make the starch granules firmer, which leads to a smaller viscosity loss of the rice flour paste at high temperatures. This is consistent with the phenomenon we observed, that high protein content corresponds to low BD (Table 3). Our RVA data support the occurrence of such restrictive effects; adding more protein increased the PeT and PaT values in RRF systems. This shows that proteins slow down the gelatinization process (Table 3). This might be because proteins slow down water absorption and particle expansion, and it takes more time and more heat for particles to break [32]. The decrease in PV, FV, and SB may be due to the fact that proteins reduce the concentration of starch and limit starch expansion. They may also physically block the rearrangement of starch sugar molecules after cooling [19,33].

With the increase in protein content in the RRF system, TV shows a nonlinear trend of first rising and then decreasing, which may indicate a transformation of the dominant mechanism. At a moderate protein level, inhibiting excessive expansion can stabilize the starch paste, prevent viscosity loss, and thereby achieve a higher TV. On the contrary, when the protein level rises, the fierce competition for water may hinder complete gelation, making the paste easier to decompose and leading to a decrease in TV [34]. It is worth noting that in our RRF model, the added protein was extracted from the same rice variety. Therefore, while the total protein content was manipulated, the fundamental protein composition (the profile of protein types) within each variety-specific RRF system remained consistent with its source. Compared to reconstituted rice flour, the gelatinization of natural starch is affected by more factors. This is why Guichao 2, even with the highest protein content (over 8%), does not show a drop in TV as protein increases. This also shows that other factors besides protein content can strongly affect how rice flour gelatinizes. This includes the components of proteins and their structural differences, the fine structure of starch granules, and the distribution of starch chain lengths, etc. These factors can improve the thermal stability of the process. These findings offer new paths for future research.

### 4.2. Proteins Influence Gel Consistency via Enhanced Thermal Stability

A central finding of this study is to clarify the potential pathway by which protein content affects gel consistency: by altering the thermal stability of the paste during the gelatinization process. The results of the relevant analysis support our view. We found that the protein content was not only strongly negatively correlated with the gel consistency (r = −0.97, *p* < 0.001), but also significantly negatively correlated with the breakdown (BD) value (r = −0.89, *p* < 0.001), indicating a connection between the two. When the protein content is relatively high, the viscosity loss of rice milk during the heating process is smaller, and the system is more stable (which also leads to a decrease in the BD value). And this improvement in stability is closely related to the decline in gel consistency.

During the heating process of starch, the higher the protein content, the lower the BD value, while trough viscosity (TV) and peak time (PeT) increase. The decrease in BD value and the increase in PeT value observed in this study support the hypothesis that “proteins limit the expansion of starch granules”. We further infer that this limiting effect may reduce the amount of amylose dissolved from the particles during the gelatinization process. It is crucial for amylose to form a continuous and elastic gel network through chain arrangement and junction formation after cooling [35,36,37]. Therefore, reducing the leaching of amylose will lead to a weaker and more fragmented gel structure, which is manifested macroscopically as a decrease in gel consistency. This hypothesis is supported by relevant analysis and microstructure: gel consistency is negatively correlated with TV and PeT (restricted swelling indicators), and positively correlated with BD (Starch granule rupture indicators). Scanning electron microscopy observation provided visual evidence: the gel with added protein showed a looser and more fragmented “honeycomb” structure, and there were obvious ungelatinized starch granules (Figure 2), provide intuitive microstructure support for the hypothesis that “proteins limit the complete gelatinization of starch”. Ungelatinized starch particles, as inert fillers, disrupt the continuity of the starch matrix [38]. In addition, the reduction in leached amylose also weakens the ability to form a strong and coherent network [39,40]. In conclusion, these pieces of evidence support a model where proteins alter the materials available for gel formation by restricting particle expansion, leading to the observed decrease in gel consistency (Figure 1).

### 4.3. Specificity in High-Amylose Background and Breeding Implications

In the high-amylose rice we looked at, the amount of amylose itself did not really show a strong connection to pasting properties or gel consistency. This indicates that in the context of high amylose content, protein may have become the main factor controlling the change in gel consistency, suggesting that protein is an important factor that cannot be ignored when choosing high-quality rice with high amylose content and processed products with high amylose content.

Gel consistency is one of the three major indicators for measuring the steamed and cooked taste quality of rice [41]. It can well represent the cooking characteristics and texture of rice, and at the same time, it can also provide an important basis for the screening of specialized rice suitable for processing. Typically, a softer gel consistency correlates with superior eating quality [42], whereas a firmer gel is often preferred for processed products such as rice noodles [41,43]. The impact of this on breeding is obvious. If the goal is to make high-amylose rice with soft gel to enhance the taste, breeders should prioritize reducing the content of grain protein. On the contrary, if the goal is to produce hard gel consistency specialty rice suitable for processing, it is recommended to seek or develop new varieties with higher protein content.

### 4.4. Study Limitations and Future Perspectives

Although our research provides convincing correlation and reconfiguration-based evidence for the strong impact of protein content on the functional properties of high-amylose rice, certain limitations should also be acknowledged. The mechanism of protein-mediated inhibition of particle expansion and interference with amylose leaching was proposed based on macroscopic viscosity measurement, correlation analysis, qualitative microstructure observation, and previous research results, rather than being directly measured. In future research, we will utilize in situ microscopy during the heating process, quantitative analysis of soluble amylose, and detailed characterization of molecular interactions between rice protein and starch components to confirm these hypotheses. In addition, environmental and genotypic factors that affect the fine structure of starch (such as the distribution of starch chain length) are not the focus of this study, but they may contribute to the observed differences in residual varieties and are worthy of further exploration.

## 5. Conclusions

Our research indicates that protein content is the main factor influencing the gelatinization behavior and gel consistency of high-amylose rice. Higher protein content is closely related to the reduction in gel consistency and the change in gelatinization characteristics. Through the reconstituted rice flour experiment, we confirmed that increasing the proportion of protein directly leads to a decrease in the consistency of the gel. Through relevant analysis and microstructure observation, we speculate that proteins may affect the formation of gel networks by enhancing the thermal stability of starch paste during heating, thereby influencing the formation of gel networks and ultimately leading to a decrease in gel consistency. This study provides an important reference for the quality breeding of high-amylose rice: when pursuing taste quality, low-protein genotypes should be selected, while high-protein germplasms can be considered when developing and processing special varieties.

## Figures and Tables

**Table 1:** Proximate Composition and Gel Consistency in Milled Rice.

Cultivar	Protein Content (%)	Gel Consistency (mm)	Crude Fat Content (%)	Total Starch Content (%)	Amylose Content (%)	Amylopectin Content (%)
Zhengui’ai	8.3 b	30 c	0.51 a	70.20 c	26.01 c	44.19 c
Guichao 2	9.1 a	27 c	0.42 b	71.95 bc	27.56 a	44.40 c
N5088	8.1 b	30 c	0.50 a	80.61 a	26.69 b	53.93 a
V5213	7.6 c	64 b	0.33 c	73.29 b	25.24 d	48.05 b
V4233	7.6 c	73 b	0.31 c	73.22 b	26.83 b	46.39 bc
Yufengzhan	7.0 d	88 a	0.21 d	70.93 c	27.17 ab	43.76 c

Note: means within a column followed by different lowercase letters are significantly different (*p* < 0.05).

**Table 2:** Pasting properties of high amylose rice cultivar.

Cultivar	PV (cP)	TV (cP)	BD (cP)	FV (cP)	SB (cP)	PeT (min)	PaT (°C)
Zhengui’ai	3856 b	2941 b	915 c	4659 c	1718 c	6.44 c	80.28 bc
Guichao 2	3944 b	3322 a	622 d	4833 b	1511 d	6.64 b	79.90 c
N5088	3637 c	3000 b	637 d	4833 b	1400 e	6.87 a	83.48 a
V5213	4191 a	3035 b	1156 b	5008 a	1972 a	6.27 d	80.73 bc
V4233	3994 b	2711 c	1283 a	4658 c	1946 a	6.17 e	81.00 b
Yufengzhan	3840 b	2547 c	1292 a	4388 d	1841 b	5.90 f	78.30 d

Note: Values within a column sharing the same lowercase letter are not significantly different ( *p* < 0.05). PV: Peak Viscosity; TV: Trough Viscosity; BD: Breakdown; FV: Final Viscosity; SB: Setback; PeT: Peak Time; PaT: Pasting Temperature.

**Table 3:** Pasting properties of RRF with different protein addition levels (0%, 5%, 10%).

RRF	Protein Content	PV (cP)	TV (cP)	BD (cP)	FV (cP)	SB (cP)	PeT (min)	PaT (°C)
Zhengui’ai	0%	4218 Aa	2236 Dc	1982 Aa	4220 Ca	1984 Ba	4.80 Dc	79.55 Ac
	5%	3259 Ab	2898 Aa	361 CDb	3637 ABb	739 Bb	6.33 Bb	81.88 Bb
	10%	2797 ABc	2550 ABb	248 BCc	3181 ABc	631 Cc	6.40 Da	82.70 Ca
Guichao 2	0%	4275 Aa	2790 Ab	1485 Ba	4842 Aa	2052 Ba	5.27 Bb	75.46 Cc
	5%	3273 Ab	3041 Aa	233 Db	3567 BCb	528 Db	6.64 Aa	84.28 Ab
	10%	2868 Ac	2671 Ab	197 Cc	3168 ABc	497 Dc	6.70 Ca	86.80 Aa
N5088	0%	3954 Ba	2467 Ca	1487 Ba	4643 Ba	2176 Aa	5.50 Ab	74.38 Cc
	5%	3100 Bb	2538 Da	562 Ab	3338 Db	800 Bb	6.97 Aa	84.30 Ab
	10%	2641 Cc	2229 Cb	412 Ac	2880 Cc	651 BCc	7.00 Aa	88.33 Aa
N5213	0%	3960 Ba	1969 Eb	1991 Aa	3662 Da	1693 Ca	4.64 Ec	79.93 Ab
	5%	2992 Cb	2498 Da	494 ABb	3457 CDb	959 Ab	5.94 Cb	81.60 Bb
	10%	2704 BCc	2450 Ba	254 BCc	3054 BCc	604 Cc	6.87 Ba	84.38 Ba
V4233	0%	3933 Ba	1966 Ec	1967 Aa	3648 Da	1682 Ca	4.67 Ec	79.60 Ab
	5%	3218 Ab	2782 Ba	437 BCb	3587 ABa	805 Bb	6.27 Bb	81.08 Bab
	10%	2761 ABCc	2450 Bb	311 Bc	3174 ABb	724 Bb	6.33 Da	81.48 CDa
Yufengzhan	0%	3937 Ba	2608 Ba	1329 Ca	4250 Ca	1642 Ca	5.00 Cc	77.90 Bb
	5%	3092 Bb	2663 Ca	429 BCb	3704 Ab	1041 Ab	6.10 BCb	79.53 Cab
	10%	2752 ABCc	2494 Bb	258 BCc	3340 Ac	846 Ac	6.37 Da	79.95 Da

Note: Uppercase letters indicate significant differences in RVA parameters between different RRF variety groups under the same protein supplementation level ( *p* < 0.05), while lowercase letters indicate significant differences in RVA parameters within the same RRF variety group across different protein supplementation levels ( *p* < 0.05).

## Data Availability

The original contributions presented in the study are included in the article. Further inquiries can be directed to the corresponding authors.

## Abbreviations

The following abbreviations are used in this manuscript:

PV	Peak Viscosity
TV	Trough Viscosity
BD	Breakdown
FV	Final Viscosity
SB	Setback
PeT	Peak Time
PaT	Pasting Temperature
SEM	Scanning Electron Microscopy
RRF	Reconstituted Rice Flour
cP	Centipoise
min	Minutes
°C	Degree Celsius
